# Containing the Threat—Don't Forget Ebola

**DOI:** 10.1371/journal.pmed.0010059

**Published:** 2004-12-28

**Authors:** Jonathan Cohen

## Abstract

In 2000, Uganda experienced the largest outbreak of Ebola fever ever described. What can we learn from the Ugandan experience to help us prepare for future outbreaks?

On 8 October 2000, the acting district director of health services for the Gulu district in northwestern Uganda received two concurrent reports of an unusual illness with high mortality, occurring in the community and at a local hospital. One report attributed the illness to poisoning at a funeral at a remote village in the far north of the district. At the same time, the medical superintendent of the hospital also reported to the health authorities that he was experiencing a cluster of cases of critically ill patients, and that there had been several deaths, including some nurses.

These events heralded what was to become the largest outbreak of Ebola fever so far described, involving 425 cases, of whom 224 died. The development of the epidemic and the measures taken to try and control it have recently been reported by Lamunu and her colleagues [1]. Their report underlines the challenges faced when dealing with such highly contagious and highly virulent infections. (At the request of *PLoS Medicine*, Lamunu et al. have made a full-text version of their report available on the World Health Organization Web site [2].)

## Ebola Virus

Ebola virus is a member of the family Filoviridae, which consists of two distinctive species, Marburg and Ebola, both of which cause severe and often fatal haemorrhagic disease in humans and monkeys. The viruses have a distinctive filamentous morphology under the electron microscope and a genome that consists of a nonsegmented, negative-stranded RNA approximately 19 kb in length.[Fig pmed-0010059-g001]

**Figure pmed-0010059-g001:**
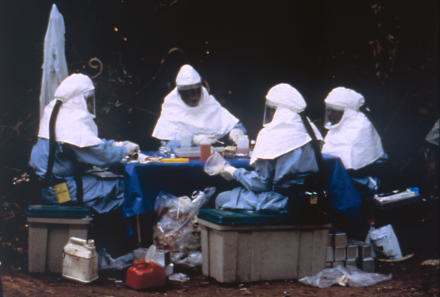
Ebola testing (Photo: Public Health Image Library, Centers for Disease Control and Prevention)

Three distinct subtypes (genotypes) of Ebola have been described that are pathogenic for humans: Ebola-Zaire, Ebola-Sudan, and Ebola–Côte d'Ivoire. A fourth type, Ebola-Reston, affects only primates but has been identified in animal facilities in the United States, Italy, and the Philippines.

## The Illness

Ebola is transmitted person to person by direct contact with infected body fluids, or by direct inoculation via contaminated instruments such as needles or razors. The incubation period of Ebola haemorrhagic fever is usually between four and 21 days. The illness is characterised by an acute onset of fever, malaise, myalgia, severe frontal headache, and pharyngitis. One of the great difficulties in making the diagnosis is that these symptoms are typical of many acute infective syndromes that occur in Ebola-endemic areas.

As the disease progresses patients develop a maculopapular rash, typically at about six days, followed by vomiting and bloody diarrhoea, with uncontrollable haemorrhage from needle sites and body orifices. Death is from shock secondary to blood loss. Treatment is largely supportive, although a recent study has reported promising results with an inhibitor of tissue factor, which may help control the bleeding diathesis [3].

## The Ugandan Outbreak

Lamunu et al. describe how initial identification of the outbreak was delayed: six weeks elapsed before the Ugandan Ministry of Health was notified. There were several reasons for this delay. In part it could be explained by a weak surveillance system, especially at the local and regional levels. But also, the nonspecific nature of the symptoms meant that the initial, sporadic cases were frequently attributed to malaria or typhoid, and patients turned to local healers for help. Important, too, was the fact that this was the first outbreak of viral haemorrhagic fever in Uganda, and lack of familiarity with the disease caused further delays.

It was only when clusters of cases became apparent that wider public health measures were instituted, and the outbreak started to come under control. In this phase, too, there were important lessons to learn. The initial identification of the disease as due to Ebola virus was made in the World Health Organization laboratories in South Africa, but soon thereafter a field laboratory was established, and this proved invaluable in guiding both case management and surveillance activities. Early involvement of specialised agencies, including the Global Outbreak and Response Network of the World Health Organization, was essential. Disseminating up-to-date information through the media, and the local communities, was important in getting the population “on side”.

## Lessons from the Outbreak

The 2000 outbreak in Uganda was the last large outbreak, but other, smaller outbreaks continue to occur. During 2004 alone there have been two further epidemics: in January there were 35 cases in the Congo, with 29 deaths, and in August a smaller outbreak in the Sudan infected 17 patients, of whom seven died [4,5]. In each of these cases the epidemic was brought under control relatively quickly, and the infection was largely localised to the immediately surrounding area. However, the lessons of the Uganda outbreak have obvious resonance with many of the recent concerns that have been raised about the global spread of infectious diseases, be they naturally acquired or related to potential biowarfare.

By and large, once an outbreak has been recognised by the public health authorities there are well-tried processes and procedures that come into play that serve to contain further spread of the infection and limit additional cases of the disease. This was shown spectacularly in the case of the SARS outbreak, in which not only was the disease controlled but the novel causative agent was identified, both within a few months. But as Lamunu and colleagues make clear, the most difficult aspect of the outbreak control is the initial recognition of the disease: diagnosis depends on the astute health-care worker who notices an unusual clinical picture, or more usually, an unexpected cluster of cases. Although the viral haemorrhagic fevers have until now been largely confined to their epidemic foci in Africa, cases will continue to occur from time to time in travellers, in whom diagnosis may be delayed.

The key lessons from the Gulu outbreak are the extremely high case mortality of Ebola and the importance of instituting rigorous procedures to control cross-infection. These lessons are crucial both for communities in Africa, where public health infrastructures are often suboptimal, and in developed countries, where the infrastructure is sophisticated but can only be deployed once the disease is recognised.
